# Uncovering Rare Structural Chromosomal Rearrangements: Insights from Molecular Cytogenetics

**DOI:** 10.3390/ijms26188886

**Published:** 2025-09-12

**Authors:** Márta Czakó, András Szabó, Ágnes Till, Anna Zsigmond, Kinga Hadzsiev

**Affiliations:** Department of Medical Genetics, Medical School, University of Pécs, 7624 Pécs, Hungary; szabo.andras@pte.hu (A.S.); till.agnes@pte.hu (Á.T.); zsigmond.anna@pte.hu (A.Z.); hadzsiev.kinga@pte.hu (K.H.)

**Keywords:** complex chromosomal rearrangement, molecular cytogenetics, chromoanasynthesis, inv dup del 18q, exceptional CCR, four-way CCR

## Abstract

Complex chromosomal rearrangements (CCRs) are rare structural abnormalities involving at least three chromosomal breakpoints and often two or more chromosomes. Owing to their inherent genomic complexity, CCRs are frequently associated with abnormal phenotypes, including developmental delay, congenital anomalies, and infertility. In this study, we report four male patients, three of them with de novo rare structural chromosomal rearrangement detected through a combination of Giemsa-Trypsin (GTG) banding, fluorescence in situ hybridization (FISH), and high-resolution microarray techniques (SNP array and array CGH). Each of the four cases turned out to be of a different type: in addition to two exceptional CCRs, an inv dup del 18q and a cluster rearrangement involving the long arm of chromosome 4 were identified. Despite the limitations of the testing methods, we performed a detailed analysis of the relationship between the most detailed genotype data and the associated phenotype. Our study provides further valuable evidence that the use of molecular cytogenetic methods is of paramount importance even in cases with abnormal karyotypes detected by light microscopy, as high-resolution data may reveal unsuspected genomic complexity, which is essential for genetic counseling in these patients.

## 1. Introduction

Complex chromosomal rearrangements were once among the least commonly observed chromosomal structural aberrations. They are extremely rare in the general population with a normal phenotype (both familial and de novo), with an incidence of about 0.003% [1]. However, their prevalence among individuals investigated for reproductive abnormalities—such as infertility, recurrent pregnancy loss, or offspring(s) with abnormal phenotype—is estimated at 0.1–0.2% [2,3].

By definition, this category includes rearrangements in which three or more breakpoints are observed on two or more chromosomes, accompanied by an exchange of genetic material between non-homologous chromosomes [4]. While fewer than 100 cases had been described in the literature by 2011, this number rose to 220 by 2018 and exceeded 380 by 2022. This rapid increase is primarily due to the widespread use of whole-genome sequencing (WGS) technologies, which—due to their high resolution—revealed and characterized previously undetected breakpoints in detail [5,6,7,8].

Currently, CCRs are classified into three categories based on the structural differences observed: type A or three-way rearrangements, which are often familial, and maternal inheritance is typical; type B or double two-way translocations, including both familial and de novo cases, often associated with Robertsonian translocations; and the most complex group, type C or exceptional rearrangements. Whereas type A and B groups are characterized by translocations only, type C group CCRs are associated with additional structural abnormalities, mostly inversions and insertions, and consequently have more breakpoints than chromosomes involved [4,9]. This group includes de novo structural aberrations, frequently with abnormal phenotypes.

Each CCR is unique; the genotype–phenotype correlation and, in particular, the mechanism of formation can only be revealed by comprehensive characterization. The technologies currently available for this purpose are the following: karyotyping as a first step can be mentioned. Due to its limited resolution, even in apparently balanced rearrangements (under a light microscope), the presence of undetected breakpoints or unbalanced regions, structural abnormalities associated with an abnormal phenotype and, therefore, clinical significance should be expected. Molecular cytogenetic techniques, such as FISH and chromosomal microarray analysis (CMA), can help in further characterization. The former is for the targeted examination of certain regions (with the limitations of available probes), and the latter is without this constraint but does not provide nucleotide-level information and cannot detect balanced rearrangements. In routine clinical diagnostics, most laboratories are equipped to apply the above conventional techniques. More detailed analysis can be expected from genome sequencing technologies [5,10]. Among genome sequencing methods, paired-end short-read sequencing is used to examine the complexities around breakpoints (the detection of breakpoints in repetitive regions is a limitation). The analysis is usually supplemented by single-molecule long-read sequencing (although, e.g., Robertsonian translocation is hard to analyze with this method). In the last few years, optical genome mapping (OGM) has become an additional option; however, it is less effective for revealing complex structural variants and does not provide nucleotide-level resolution. Validation of any detected breakpoints is most often performed by Sanger sequencing. Unfortunately, both OGM and genome sequencing technologies are very expensive and time-consuming, and access to the technology is limited, particularly in the case of OGM. The entire methodological spectrum described above is generally only available for research purposes in suitable laboratories. Numerous high-quality studies have been reported in the literature on the application of these advanced techniques in individual CCR cases [5,6,7,8,10,11,12].

In the present manuscript, we report four cases with rare chromosomal rearrangements analyzed over a 26-year period in the clinical diagnostic laboratory of our department. These include one prenatal and three postnatal cases, supplemented by the assessment of genotype–phenotype correlations. As the analyses were performed at different time points, we applied the most advanced diagnostic technologies available at each respective time to achieve the most accurate characterization of the rearrangements possible.

## 2. Results

### 2.1. Patient 1

#### 2.1.1. GTG Banding

**Figure 1:**
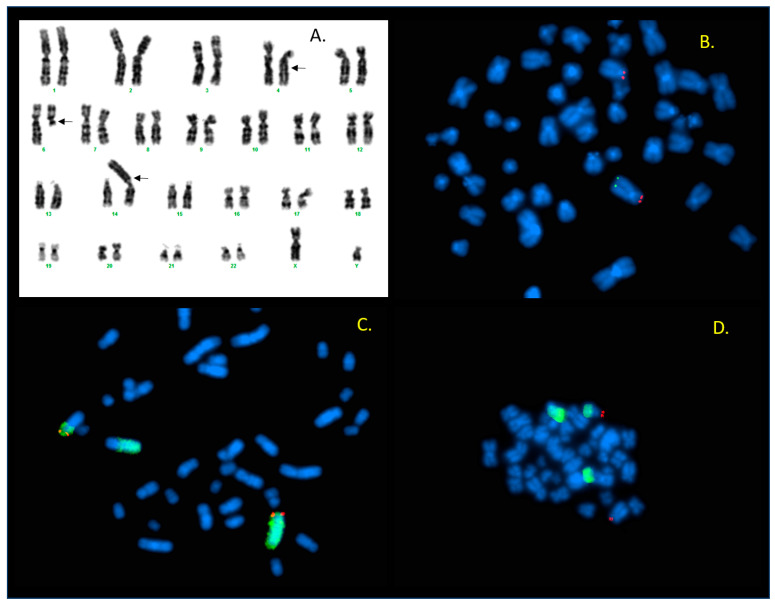
G-banding and FISH results of Patient 1. (**A**) Karyogram of Patient 1, showing a rearrangement involving chromosomes 4, 6, and 14. Arrows indicate the breakpoints of translocation. (**B**) Subtelomeric probes specific to 4p (red)/4q (green): subtelomeric 4q is present only on intact chromosome 4. (**C**) Signals obtained by combining the whole chromosome 4 painting (WCP) probe (green) with the 4p subtelomeric probe (red) show that the 4p signal is detectable on derivative chromosome 4, while the long arm of der(4) shows no green hybridization signal (left). The missing segment of 4q can be seen translocated to chromosome 14 (in the middle). (**D**) Hybridization pattern of 4p subtelomeric probe (red) and WCP 6 probe (green): 4p signal is present on both the intact and the derivative chromosome 4. The long arm of der(4) shows the green signal of WCP 6.

Metaphase karyotypes revealed complex structural abnormalities involving three chromosomes, such as chromosomes 4, 6, and 14 (Figure 1A) (Supplementary Figures S1–S4). Unfortunately, parental samples were not available for analysis, so it is not known whether the structural changes were inherited or occurred de novo.

#### 2.1.2. FISH

**Figure 2:**
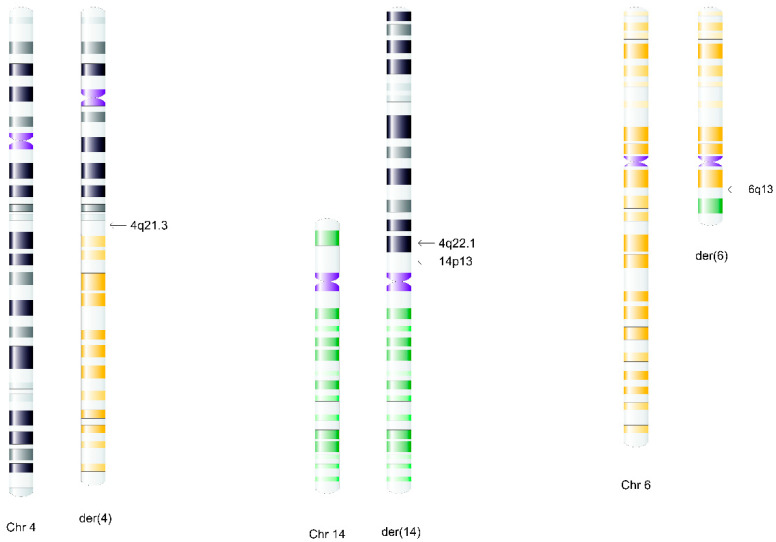
Schematic illustration of the rearrangement in Patient 1. The broken long arm of chromosome 6 (yellow) is translocated to 4q (gray/black). However, the broken 4q moved to the terminal short arm of chromosome 14 (green). The short arm of chromosome 14 can be seen translocated to the broken end of 6q. (The centromeres are marked in purple).

FISH studies were performed using WCP probes specific to chromosomes 4, 6, and 14, as well as subtelomeric probes for the same chromosomes (manufacturer: Vysis, Abbott, Chicago, IL, USA). The results indicated that the missing segment of the long arm of chromosome 6 was translocated to the long arm of chromosome 4, and the broken 4q segment was subsequently transferred to the short arm of chromosome 14 (Figure 1C,D). The subtelomeric signal for the short arm of chromosome 4 showed a normal pattern, excluding a 4p terminal deletion (Figure 1B). The abnormality of the short arm of chromosome 4, as shown in the karyogram (Figure 1A), is presumably due to multiple breaks along chromosome 4 and inversion of the segments between the breakpoints. This hypothesis could not be confirmed or ruled out by our results. In summary, FISH analysis identified a deletion in the 4q terminal region only. Figure 2 shows a schematic representation of the rearrangement. The karyotype can be described as 46,XY,t(4;14;6)(q21.3;p13;q13).ish der(14)t(4;14;6)del(4)(q35).

### 2.2. Patient 2

#### GTG Banding and FISH

**Figure 3:**
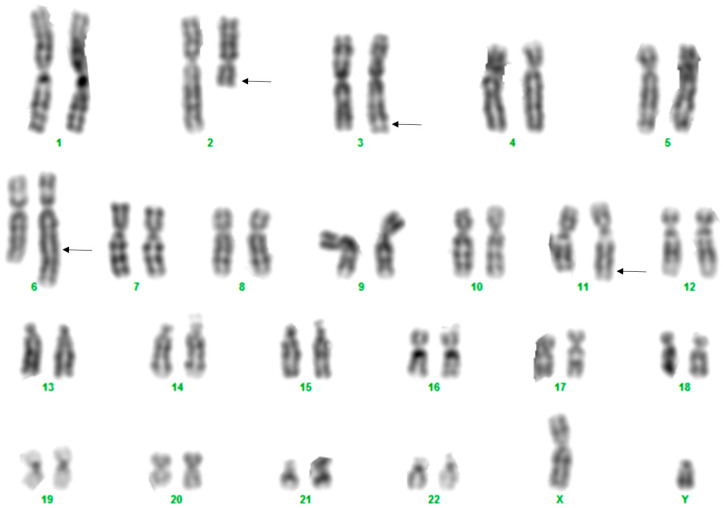
Karyogram of a cultured fetal cell of Patient 2. Arrows indicate the translocated segments detected by FISH analysis.

**Figure 4:**
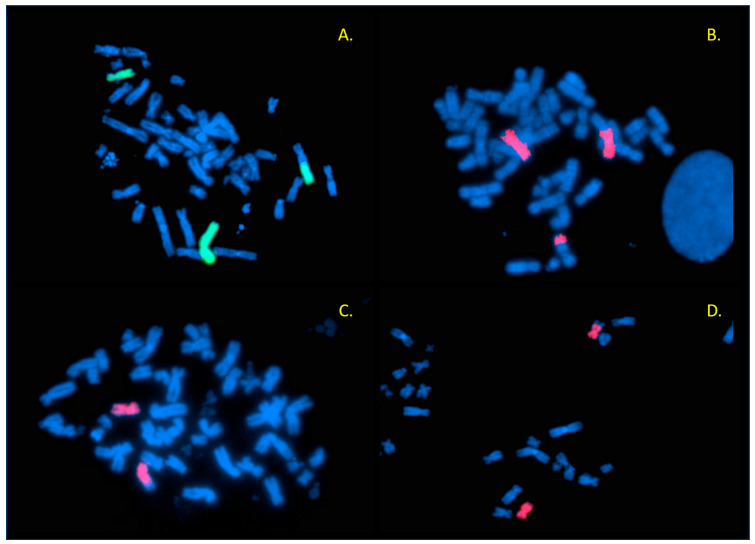
Hybridization signals of the WCP probes (Patient 2). (**A**) WCP 2 probe (green) demonstrates the translocated 2q on the long arm of der(6) (right). (**B**) WCP 6 probe (red): der(6) can be seen on the right side, the translocated segment of 6q can be detected at the distal end of 11q (bottom). (**C**) WCP for chromosome 11 (red) clearly shows that the probe does not give a signal at the terminal part of the long arm of der(11) (bottom). (**D**) WCP 11 (red) with der(11) at the top of the picture.

**Figure 5:**
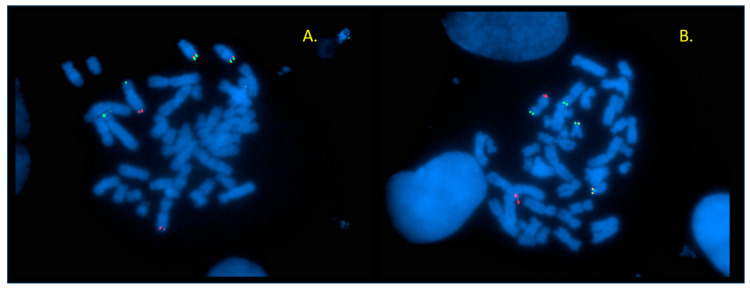
Results of subtelomeric FISH probes (Patient 2). (**A**) The 6p signals (green) are present on both the intact and derivative chromosome 6 (left side), and 6q (red) is missing on der(6), but it is translocated to 11q (bottom). There are two acrocentric chromosomes (chrs 13) at the top of the picture with dual color signals on the subtelomeric region of 13q (this was part of the probe mixture used). (**B**) Subtelomeric 11p (green) signals are present on der(11) (at the top of the metaphase, near the intact chromosome 11); on the other hand, 11q (red) can be detected on the distal long arm of der(3) (lower left part of the metaphase). Dual color signals can be observed on 18p as part of this probe mixture.

**Figure 6:**
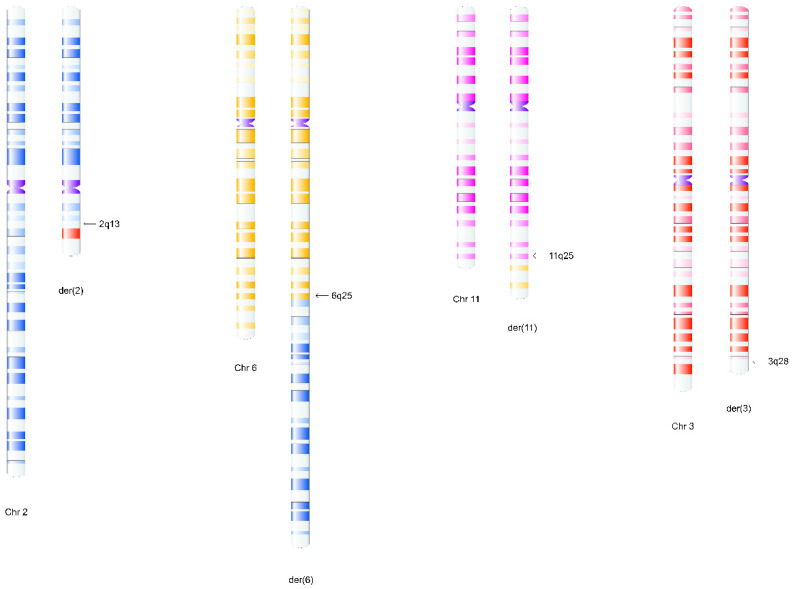
Illustration of the complex rearrangement involving chromosomes 2, 6, 11, and 3 in Patient 2.

The fetal karyotype clearly revealed structural rearrangements involving four chromosomes: 2, 3, 6, and 11 (Figure 3) (Supplementary Figures S5 and S6). Given the ongoing pregnancy, FISH analysis was primarily aimed at determining whether the rearrangement was balanced or unbalanced. However, FISH analyses performed (using WCP and subtelomeric probes specific to the chromosomes that have undergone rearrangements; manufacturer: Vysis, Abbott) demonstrated that this was not the coexistence of two independent reciprocal translocations, but rather a complex rearrangement involving the simultaneous exchange between the four chromosomes as a single process. Specifically, the broken part of the long arm of chromosome 2 was replaced on the long arm of chromosome 6; the 6q terminal region was translocated on 11q; the 11q terminal segment was moved to the terminal end of 3q; and the missing 3q fragment completed the loop by joining the broken end of the long arm of chromosome 2 (Figure 4A–D and Figure 5A,B, Supplementary Figures S12–S16). In summary, the karyotype is as follows: 46,XY,t(2;6;11;3)(q13;q25;q25;q28) (Figure 6). Based on the chromosome analyses of the parents, the rearrangement in the fetus proved to be de novo.

### 2.3. Patient 3

#### 2.3.1. GTG Banding

Karyotyping revealed a terminal deletion on the long arm of chromosome 18: 46,XY,del(18)(q21) (karyogram: Supplementary Figures S7–S10). Chromosomal analyses of the parents showed no abnormalities, suggesting that the 18q deletion was de novo.

#### 2.3.2. Array CGH

**Figure 7:**
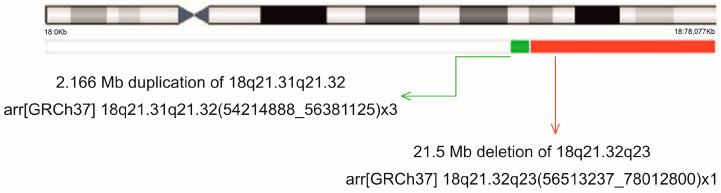
Schematic representation of the 18q duplication and deletion detected in Patient 3 by array CGH.

Despite the established chromosomal diagnosis, array comparative genomic hybridization (array CGH) was requested by the clinical geneticist due to the patient’s phenotype. The array result confirmed a 21.5 Mb terminal deletion at 18q and identified the deleted region’s breakpoints as 18q21.32q23. Additionally, a 2.166 Mb duplication proximal to the deletion, separated by a disomic spacer (132.1 kb), was identified: arr[GRCh37] 18q21.31q21.32(54214888_56381125)x3,18q21.32q23(56513237_78012800)x1 (Figure 7).

### 2.4. Patient 4

#### 2.4.1. GTG Banding

Patient 4 was referred to our department following a normal karyotype result. Chromosome analyses of the parents were performed after the child’s array investigation and revealed no abnormalities in either of them.

#### 2.4.2. SNP Array

**Figure 8:**
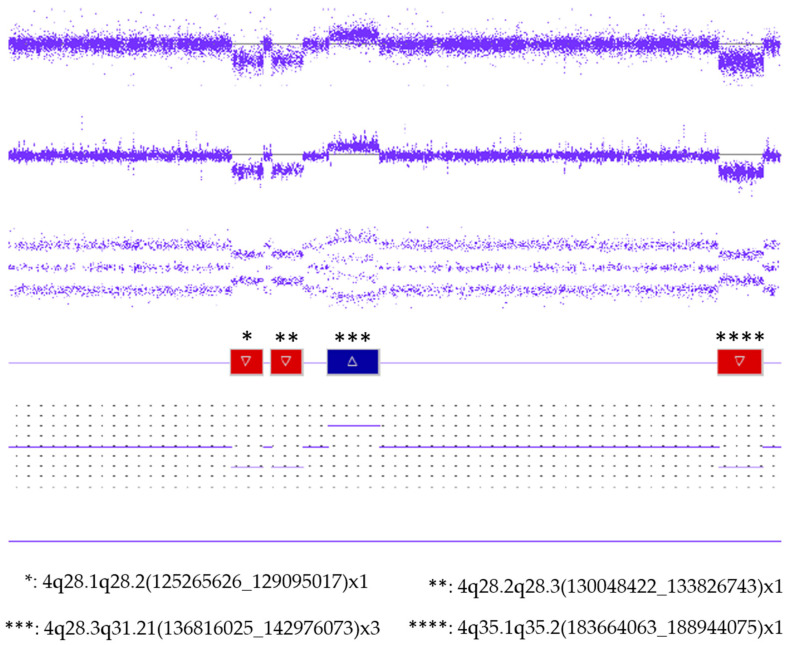
SNP array results of Patient 4. Array image of the 4q terminal region shows three deletions, one duplication, and the disomic segments separating them.

CMA results identified four copy number variations on the long arm of chromosome 4: three deletions (3.829 Mb, 3.778 Mb, and 5.280 Mb in size, respectively) and one duplication (6.160 Mb). All of them were located in close proximity to each other (Supplementary Figure S11): arr[GRCh37] 4q28.1q28.2(125265626_129095017)x1,4q28.2q28.3(130048422_133826743)x1,4q28.3q31.21(136816025_142976073)x3,4q35.1q35.2(183664063_188944075)x1. The enlarged image clearly shows that the duplication and the three deletions are separated by disomic regions (Figure 8).

## 3. Discussion

In this study, we describe four cases with rare chromosomal rearrangements identified during diagnostic examinations over the past 26 years—three postnatally and one prenatally. Due to the limited technological capabilities available at the time, only GTG banding and FISH were performed in the first two cases. In contrast, cases 3 and 4 were investigated using CMA, which provided additional insights.

Karyotype and FISH studies performed on the sample of Patient 1 revealed the involvement of three chromosomes, 4, 6, and 14, respectively, in the structural rearrangement (Figure 1). Although only three chromosomes were implicated, the number of chromosomal breakpoints is likely higher. Since a terminal deletion was not confirmed on the short arm of chromosome 4, which appears to be shorter, the observed difference can be explained by multiple intrachromosomal breaks and the inversion of the segments between the breakpoints. The only unbalanced condition that could be detected by FISH at that time was the deletion of the terminal region of 4q. Unfortunately, based on our studies, neither the presence of additional breakpoints suspected on chromosome 4 can be confirmed nor can the size of the detected 4q terminal deletion be assessed. Comparing the child’s phenotype with the clinical features of patients with 4q terminal deletion, it can be stated that severe growth and psychomotor delay, brachycephaly, minor craniofacial dysmorphism including a flat nose, hypoplastic midface, low-set rotated ears, the observed strabismus, and generalized muscle hypotonia correspond to the 4q minus syndrome. Nonetheless, the absence of characteristic signs such as ocular malformations, cardiac or genitourinary anomalies, skeletal abnormalities, gastroesophageal reflux, orofacial clefts, or the distinctive fifth finger/nail configuration suggests phenotypic variability. The latter are known in the majority of patients with terminal 4q deletion, but the other symptoms above can vary greatly. Most patients with 4q deletion have psychiatric symptoms like aggression, verbal hallucinations, mood swings, and delusions, which are not observable in our patient. However, the child was last examined at the age of 4 years, so we have no information about his symptoms at an older age. Since the child was placed in residential care at the age of one, we did not have access to parental samples, and thus, the inheritance status remains unknown.

The examinations of Patient 2 were carried out during his fetal age. Our information regarding the phenotype was limited to the results of ultrasound examination performed during the pregnancy; furthermore, the results of prenatal screening tests were available, which did not indicate any abnormality related to an increased risk of chromosomal abnormalities. It is important to emphasize that the indication for amniocentesis was an advanced maternal age (38 years). Karyotyping clearly indicated a structural rearrangement involving four chromosomes (2, 3, 6, and 11, respectively) (Figure 3). Our FISH studies detected no gain or loss of genetic material, although this cannot be excluded based on the results of the WCP and subtelomeric probes used. It may be assumed that the complex rearrangement in this case would be the result of a simultaneous event (Figure 6). This de novo CCR may be considered as balanced mainly because the child was born at term, exhibited no clinical abnormalities at birth, continues to develop normally at age seven, and attends a normal school. Based on the literature data, infertility and subfertility are predominant characteristics in balanced CCR carrier male patients, and pregnancy outcomes associated with such cases are typically poor, with spontaneous pregnancies being very rare [13,14,15,16]. This is probably due to a disturbance occurring during gametogenesis in CCR-carrying males [4], caused by the presence of the chromosomal rearrangement altering synapsis formation in the prophase of meiosis I, leading to the arrest of spermatogenesis through several mechanisms (such as failure of synapsis, heterosynapsis, association of asynaptic segments with the X-Y body altering the X-inactivation process, or altering the transcription of genes necessary for successful completion of meiosis). Based on all these observations, the development of a fertility disorder in adulthood in Patient 2 cannot be excluded.

**Figure 9:**
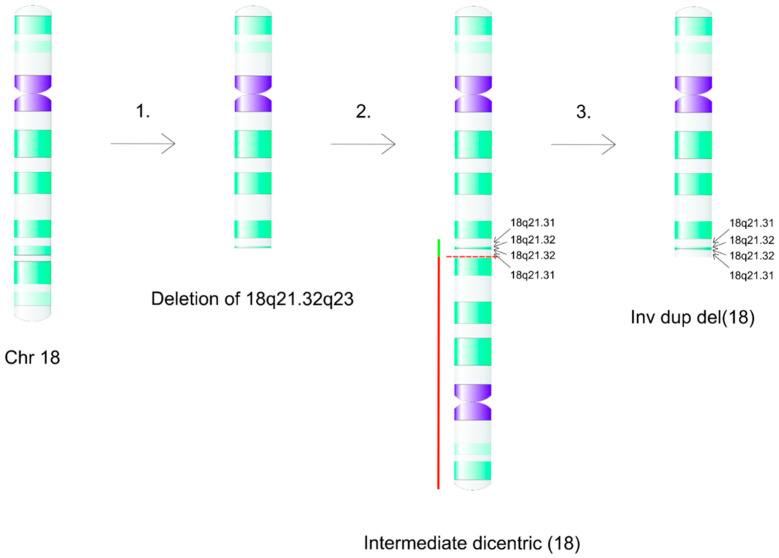
The mechanism of formation of inv dup del 18q. 1: the first step is the formation of a double-stranded DNA break at 18q21.32, leading to a terminal deletion (18q21.32q23). 2: By activation of the repair mechanism, a dicentric intermediate (18pter→18q21.32::18q21.32→18pter) is established with fusion of the broken ends. The presence of the two centromeres makes this structure unstable during cell division. 3: The dicentric chromosome breaks repeatedly, so then two monocentric reciprocal chromosomes (one of them has a terminal deletion, the other an inverted duplication and terminal deletion) develop (BFB cycle). This process ends with the formation of a new telomere.

The deletion of the terminal segment of the long arm of chromosome 18 was clearly visible on the karyotype of Patient 3. Based on the clinical symptoms, the array CGH study was performed, which confirmed a more complex structural abnormality (Figure 7), which is called inverted duplication with terminal deletion (inv dup del). The known mechanism of its development is a double-stranded DNA break that results in a terminal deletion, in our case with the breakpoint of 18q21.32. By repairing the double-stranded DNA breakage, a temporary dicentric intermedier (18pter→18q21.32::18q21.32→18pter) is formed by the fusion of the ends of the fragments, which makes this structure unstable during cell division (Figure 9). Since in anaphase it may be pulled towards two opposite poles of the cell, it can break, resulting in two monocentric reciprocal chromosomes (one of them has a terminal deletion, the other an inverted duplication and terminal deletion). This process is called the breakage–fusion–bridge (BFB) cycle, and as a final step, the broken end of the new chromosome is stabilized by the formation of a new telomere. By application of new characterization technologies, it has become known that the formation of dicentric intermediates can occur in four ways. These can be identified based on the characteristics of the mechanism that actually repairs the double-strand break (NHEJ or NAHR: non-homologous end joining and non-allelic homologous recombination) and the presence or absence of the disomic spacer separating the inverted regions [17,18,19,20,21,22,23]. Given that the array CGH we used, in addition to breakpoint identification, confirmed the presence of a disomic spacer with a size of 132.112 kb, we can also exclude one of the four mechanisms from the list of possibilities: the U-type exchange. Unfortunately, in the absence of sequencing data, it cannot be determined which of the remaining three mechanisms created the dicentric intermediate in our case. The most likely of the known possibilities is the so-called fold-back mechanism [21], which creates a monocentric chromosome through intrastrand pairing within a microhomology region. The dicentric intermediate can then form during DNA replication. This theory is supported by the large number of intrachromosomal segmental duplications on chromosome 18 (according to data from the UCSC genome browser, there are 24 segmental duplications in the terminal band 18q23, one in the 18q21.31 region, and another in the 18q21.32 band). Due to the complex structure that has been produced, the functioning of MMBIR/FoSTES (Microhomology-Mediated Break-Induced Replication)/(Fork Stalling and Template Switching) mechanisms is also likely during DNA replication. The case we examined shows a very interesting similarity to that presented in the recently published paper by Bonaglia et al. [24]. Here, the authors describe an inv dup del 18q structural abnormality (in addition to the presence of two 18q deletions of different sizes) in a 5-year-old girl, where the 2.7 Mb inverted duplication segments are separated by a 4.7 kb disomic spacer, followed by a 21 Mb sized deletion. The breakpoints in the patient we examined are very similar to those described by Bonaglia et al. Analyzing the genomic content of the duplicated region, 16 protein-coding genes can be found, and 10 of them are listed in OMIM (*ATP8B1* MIM # 602397, *NEDD4L* MIM # 606384, *MALT1* MIM # 604860, *TXNL1* MIM # 603049, *FECH* MIM # 612386, *NARS1* MIM # 108410, *WDR7* MIM # 613473, *ST8SIA3* MIM # 609478, *ONECUT2* MIM # 604894, *ALPK2* MIM # 619965). Based on the ClinGen database, none of these has a triplosensitivity score of ≥0.94, so their contribution to the child’s phenotype cannot be proven.

The boy we examined and the girl reported by Bonaglia et al. have very similar inv dup del 18q structural chromosome abnormalities, so it may be interesting to compare the associated phenotypes. Although this structural chromosome abnormality is known to have a variable phenotype, there are characteristic clinical symptoms that are shared by the majority of those affected. Some of these can be seen in both children, such as delayed psychomotor development, seizures, absent speech, generalized hypotonia, short stature, poor weight gain, and microcephaly. There are some features that are only observed in one of the two children: congenital aural atresia and deafness, as well as eye movement disorder, congenital heart defect, and joint laxity, only occurred in the case of Bonaglia et al., whereas scoliosis, clubfeet, hypopigmentation, hyperactivity, features of autism spectrum disorder, and genital hypoplasia are only observed in Patient 3. Growth failure and intellectual disability are much more pronounced in our patient. Brain malformation cannot be detected in either of the children. With regard to endocrine features, it is interesting to note that insulin-like growth factor-1 levels in the young girl are within the lower range of normal, whereas in Patient 3, the only detectable endocrine abnormality is hyperthyroidism, which is less characteristic in similar patients, with hypothyroidism being more typical. In our opinion, the most interesting finding in the comparison is the cleft palate symptom. In their publication, Bonaglia et al. suggest a causal role for the *NEDDL4* (MIM # 606384) and *TSHZ1* (MIM # 614427) genes; the first one is located in the duplicated 18q genomic region, and the second one is located in the deleted 18q22.3 band. Both genes are similarly affected in Patient 3. In our case, although there is no clear cleft palate observable, there is a narrow palate among the features, so the similar copy number of the two genes is indeed thought-provoking.

The chromosomal structural abnormality observed in Patient 4 is completely different from the rearrangements detected in the three cases presented above. The copy number variations (CNVs) revealed by the SNP array performed (three deletions and one duplication) are located in close proximity to each other on the long arm of chromosome 4 (Figure 8), separated by normal copy number regions. This pattern of CNVs, involving one chromosome arm and containing deletions and duplications as well, suggests chromoanasynthesis as the most likely formation mechanism [25,26,27,28]. During this process, a part of a chromosome breaks into DNA segments, followed by a re-synthesis and joining of them using replication-based mechanisms such as FoSTeS and MMBIR [29,30,31,32,33,34]. Unfortunately, in the absence of a sequence analysis of the breakpoints, we cannot come any closer to the precise mechanism of formation.

Regarding the genomic content of the CNVs detected in Patient 4, the most proximal 4q deletion (4q28.1q28.2(125265626_129095017)x1) can be highlighted because of the *MFSD8* gene (MIM # 611124, MAJOR FACILITATOR SUPERFAMILY DOMAIN-CONTAINING PROTEIN 8), which may be prominent in terms of genotype–phenotype correlation. This is associated with neuronal ceroid lipofuscinosis 7, a disease that was suspected based on the specific signs of our patient’s brain MRI scan. However, the inheritance of the disease is autosomal recessive (AR). Infantile neuronal ceroid lipofuscinosis (NCL) is a lysosomal storage disease that results in progressive dysfunction of the central nervous system (CNS) and neurodegeneration. The classic early infantile onset NCL has a heterogeneous genetic background involving different genes, including *PPT1* (lysosomal lipid hydrolase, MIM # 600722), *CTSD* (cathepsin D, lysosomal peptidase, MIM # 116840), and *KCTD7* (a member of the KCTD gene family with a potassium channel tetramerization domain, MIM # 611725). Infants usually present with encephalopathy, epilepsy, microcephaly, and developmental delay, which is not equivalent to the phenotype of Patient 4. However, among the genes associated with the late-infantile onset type of NCL, besides the lysosomal exopeptidase *TPP1* (MIM # 607998), the role of *MFSD8* gene coding for a transmembrane protein is also known. Given the AR inheritance of the disease, in the case of a heterozygous deletion, the sequence data of the homologous allele would be required to support a causal association with the phenotype, but no such study has been performed. There is no known evidence supporting the haplosensitivity for the *MFSD8* gene in the ClinGen database. Regarding the other three identified CNVs, two distal deletions, and the duplication between them, it can be ascertained that these do not provide any additional information in terms of genotype—phenotype correlation.

As mentioned above, our institute’s laboratory performs clinical diagnostics. The cases presented in the manuscript were examined at different times over the past 26 years. We used the best technical capabilities available to us at the time for each patient’s examination. Nevertheless, it is important to note that the examination of the four cases presented in this manuscript is not without limitations. If it had been possible at the time, we would have supplemented the examination of Patient 1 with array CGH to determine whether the terminal 4q deletion detected was the only imbalance. Based on the clinical symptoms, it would also have been worth ruling out uniparental disomy of chromosomes 6 and 14. Unfortunately, in the absence of a DNA sample, we were unable to do this retrospectively. Furthermore, if we were to examine Patient 2’s sample today, we would perform array CGH to confirm the balanced chromosomal state. In Patient 3, FISH probes specific for the deleted and duplicated 18q regions could visually display and confirm the structure determined based on the results of the array CGH test. Finally, in Patient 4’s case, optical genome mapping or region-specific FISH tests could yield extremely valuable information, but unfortunately, we do not have the financial resources available for these. Nevertheless, we considered it worthwhile to present the rare chromosomal structural abnormalities described in the manuscript.

## 4. Materials and Methods

### 4.1. GTG Banding

Giemsa–Trypsin banding was performed on peripheral blood lymphocytes using the standard procedure for karyotyping [35]. In the case of Patient 2, chromosome analysis was performed on cultured cells obtained by amniocentesis.

### 4.2. FISH

To investigate the individual cases, a combination of WCP and subtelomeric probes specific to each chromosome involved in the rearrangement was used (Vysis, Abbott Laboratories, Abbott Park, IL, USA; Leica Biosystems, Nussloch, Germany) [36].

### 4.3. Array CGH

Array CGH [37] was performed using the Agilent Human Genome Sureprint G3 8x60K oligo-array (Amadid 021924) (Agilent, Santa Clara, CA, USA), following the manufacturer’s protocols. The CGH array of Patient 3 occurred using the BlueGnome 8x60K platform, which was then produced by Agilent. DNA extraction from peripheral blood leukocytes occurred using the E.Z.N.A.^®^ Blood DNA Maxi kit (Omega BIO-TEK, Norcross, GA, USA), in accordance with the manufacturer’s instructions. A NanoDrop 2000 spectrophotometer (Thermo Fisher Scientific, Waltham, MA, USA) was applied for measuring the concentration and purity of extracted DNA. Data were processed using BlueFuse Multi software (v 4.0). Genomic coordinates refer to the UCSC Genome Browser (GRCh37/hg19).

### 4.4. SNP Array

The Applied Biosystems™ CytoScan™ 750K Accel Array (Thermo Fisher Scientific Inc., Waltham, MA, USA) was used for high-resolution genome-wide copy number analysis. The sample tested was genomic DNA from peripheral blood leukocytes, isolated as described above (*4.3. Array CGH*). Laboratory procedures followed the manufacturer’s recommendations. For analysis of the results, the Applied Biosystems™ Chromosome Analysis Suite, v.4.5.0.34. software (Thermo Fisher Scientific, Inc., Waltham, MA, USA) was used as recommended by the manufacturer. Similarly to the above, genomic annotations were referenced to the UCSC Genome Browser (GRCh37/hg19).

### 4.5. Case Presentations

#### 4.5.1. Patient 1

The patient was born at term from the mother’s first pregnancy, with a birth weight of 3450 g (75th percentile), a length of 51 cm (25th percentile), and a head circumference of 31 cm (10th percentile). The parents were apparently healthy, and both family and pregnancy histories were unremarkable. The patient exhibited brachycephaly, a full periorbital region, curved eyebrows, double eyelashes, strabismus, a flat nose, and midface hypoplasia. Neurological examination detected generalized muscle hypotonia. Cranial and renal ultrasound showed no abnormalities; cardiac echocardiography and fundus examination were also normal. The child was placed in residential care at one year of age. At that time, he exhibited severe growth and psychomotor delay; his height, weight, and head circumference were below the third percentile, and his developmental quotient (DQ) was 42. He was unable to roll over, hold up his head, or babble. Chromosome analysis was indicated due to craniofacial anomalies and muscle hypotonia.

#### 4.5.2. Patient 2

The fetus was at 17 weeks of gestation when the mother underwent amniocentesis due to advanced maternal age. There was no family history of chromosomal abnormalities, and the pregnancy was uneventful. Fetal screening tests indicated a risk appropriate to maternal age, and ultrasound findings were normal. Despite the identification of complex chromosomal rearrangements during prenatal karyotyping, the parents decided to continue the pregnancy. A healthy infant was born at term. According to parental reports, the child is healthy and attends a regular school.

#### 4.5.3. Patient 3

The child was born at 38 weeks, with a birth weight of 2780 g (25–50th percentile) and Apgar scores of 10/10. Abdominal and cranial ultrasounds were normal. Family history was unremarkable. The mother’s previous pregnancy ended in spontaneous abortion at 10 weeks. At the age of 3.5 years, the child was referred for genetic evaluation due to severe growth deficiency: weight: 11,170 g (<3rd percentile), height: 85 cm (<3rd percentile), and head circumference: 46.5 cm (<−2 SD). Clinical features included scoliosis, clubfeet, hypopigmentation, bruxism, and palmar hyperkeratosis. Genital anomalies included hypoplasia, hypoplastic scrotum, and cryptorchidism. Dysmorphic features were observed, among them, microcephaly, microphthalmia, deep-set eyes, short philtrum, and narrow palate. Neurological examination revealed generalized hypotonia and delayed psychomotor development. Speech was absent. He had stereotyped hand movements (wiping and hand washing), hyperactivity, and poor articulation. Short attention span and absence of eye contact were characteristic. Investigations (cranial/abdominal ultrasound, EEG, hearing, ophthalmology) were normal. At 7 years, he could walk independently with difficulties. At the age of 8 years, seizures were observed, and hyperthyroidism developed. Valproate therapy significantly reduced seizure frequency. Severe psychomotor delay was confirmed.

#### 4.5.4. Patient 4

The patient was born at 38 weeks of gestation. The mother’s previous pregnancy ended in spontaneous abortion at the 11th gestational week. The birth weight was 3940 g (75–90th percentile), with Apgar scores of 7/8. Elevated CSF protein levels were detected; TORCH infection tests were negative. Abdominal ultrasound showed hepatomegaly, and cardiac evaluation identified VSD, FoA, PDA, and cardiomegaly. EMG showed no abnormalities, and ophthalmology was negative as well. A cranial MRI scan raised the possibility of infantile cerebral ceroid lipofuscinosis or gangliosidosis. Later, the possibility of gangliosidosis was excluded. His motor development was delayed with significantly reduced muscle tone. His speech development was delayed with some words at the age of 2 years. Speech and language therapy began at the age of four. He was under ophthalmological care for astigmatism. Informative morphogenetic variants were relative macrocephaly, high forehead, hypertelorism, midface hypoplasia, deep nasal root, short nose, low-set dysplastic ears, micrognathia, narrow palate, short neck, hypoplastic right thorax, shortened limbs, and flat feet.

## 5. Conclusions

In summary, the karyotyping and molecular cytogenetic studies performed in our institute over the decades enabled the identification and characterization of rarely observed complex chromosomal rearrangements in four patients. Three of them were confirmed de novo; however, in Patient 1, the inheritance is unknown. The only prenatally detected CCR was apparently balanced, and the three postnatal cases proved to be unbalanced. These observations are consistent with the data known so far, such as the CCRs described in the literature, which consist of 70–75% de novo cases, of which about half are associated with an abnormal phenotype [38,39]. Unfortunately, without whole-genome sequencing, it was not possible to determine the precise breakpoints and the mechanism of formation in our cases. Despite the limitations of the methods used, our study contributes to the cases already published in the scientific literature by presenting four more rare structural abnormalities, two CCRs, an inv dup del 18q, and a chromotripsis-like rearrangement. The four cases presented provide further evidence that the use of molecular cytogenetic methods can provide additional information even in the presence of an abnormal karyotype; moreover, it may reveal an unexpected level of genomic complexity. This information is of crucial importance for genetic counseling. Our findings highlight the importance of integrated cytogenetic and molecular analyses in the diagnosis and characterization of rare structural chromosomal abnormalities and emphasize the relevance of combining chromosome- and genome-level information to understand the genotype–phenotype correlation in affected individuals.

## Data Availability

The original contributions presented in this study are included in the article/supplementary material. Further inquiries can be directed to the corresponding author.

## Supplementary Materials

The following supporting information can be downloaded at: https://www.mdpi.com/article/10.3390/ijms26188886/s1.

## Abbreviations

The following abbreviations are used in this manuscript:

CCR	complex chromosomal rearrangements
array CGH	array comparative genomic hybridization
FISH	fluorescence in situ hybridization
GTG banding	Giemsa–Trypsin banding
SNP array	single-nucleotide polymorphism probe-containing array
WGS	whole-genome sequencing
CMA	chromosomal microarray analysis
OGM	optical genome mapping
BFB	breakage–fusion–bridge cycle
NHEJ	non-homologous end joining
NAHR	non-allelic homologous recombination
CNV	copy number variation
FoSTeS	Fork Stalling and Template Switching
MMBIR	Microhomology-Mediated Break-Induced Replication
UCSC	University of California, Santa Cruz, Genome Browser database
EEG	electroencephalography
GRCh37	Genome Reference Consortium Human Build 37
WCP	whole chromosome painting FISH probe
