# Supplementary figures and images for: Uncovering Rare Structural Chromosomal Rearrangements: Insights from Molecular Cytogenetics
Source: Int J Mol Sci. 2025 Sep 12;26(18):8886. doi: 10.3390/ijms26188886 (PMC12469836; doi:10.3390/ijms26188886)

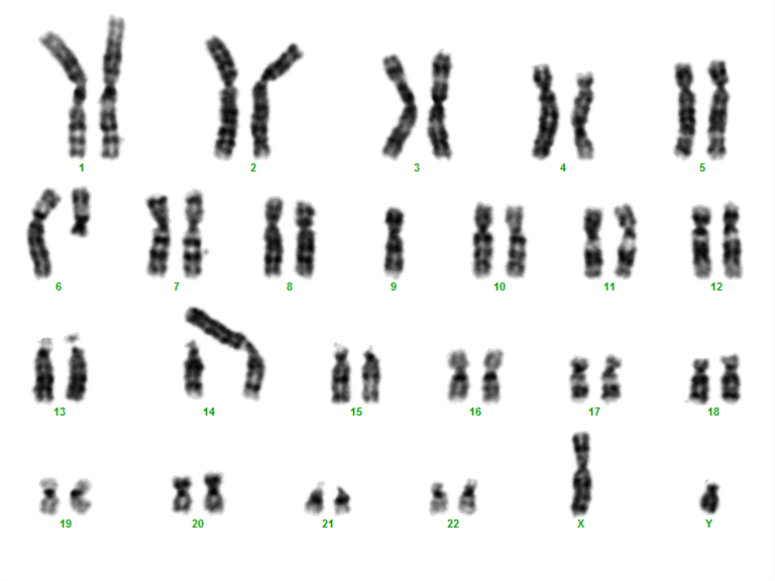

Supplement: Supplementary file 1 [file ijms-26-08886-s001.zip › Supplementary Figure 1.tif]

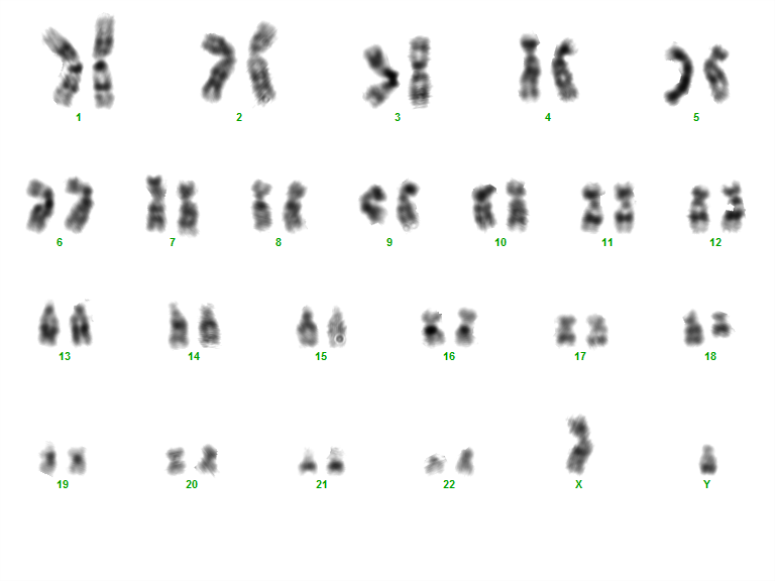

Supplement: Supplementary file 1 [file ijms-26-08886-s001.zip › Supplementary Figure 10.tif]

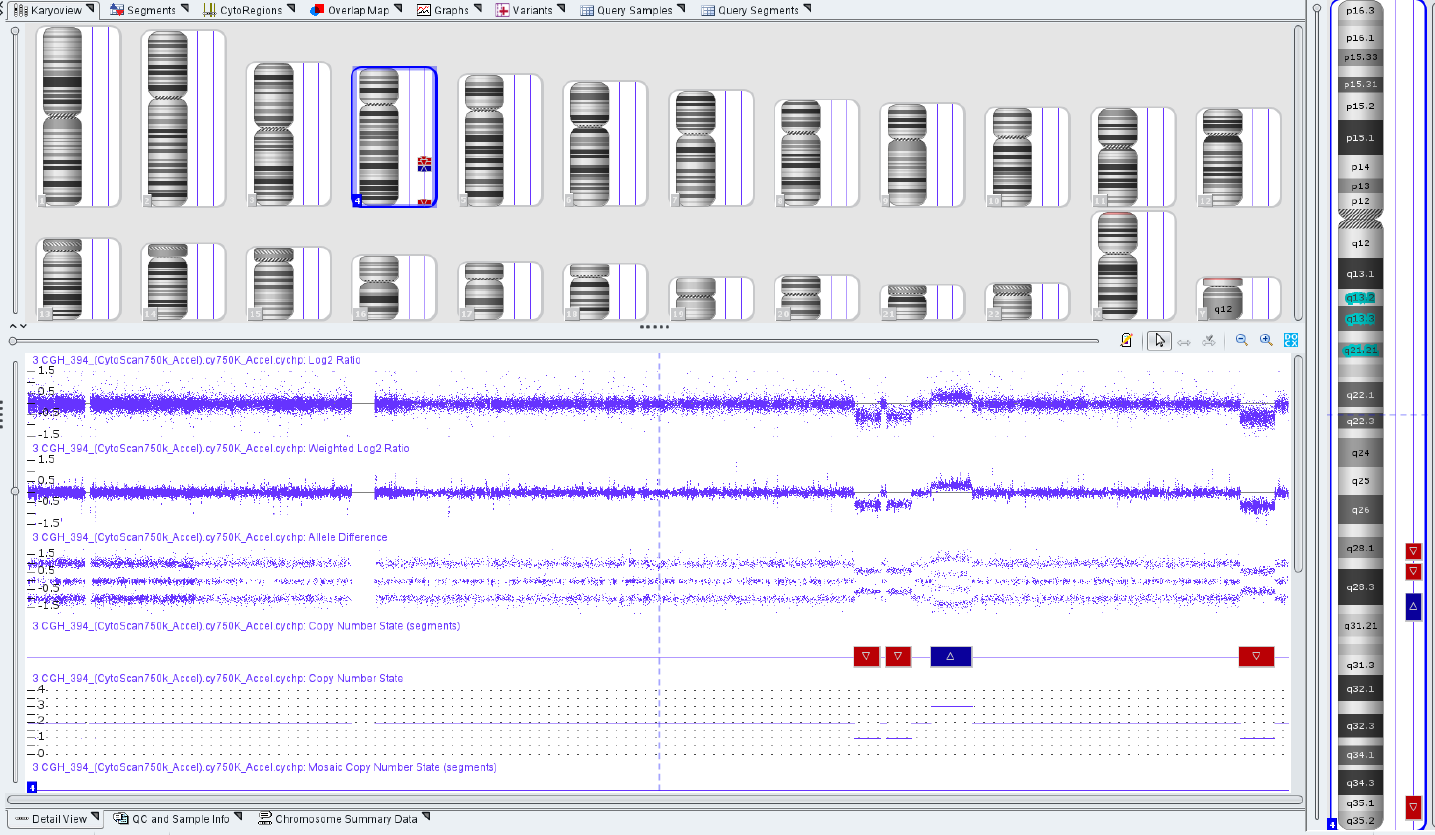

Supplement: Supplementary file 1 [file ijms-26-08886-s001.zip › Supplementary Figure 11.PNG]

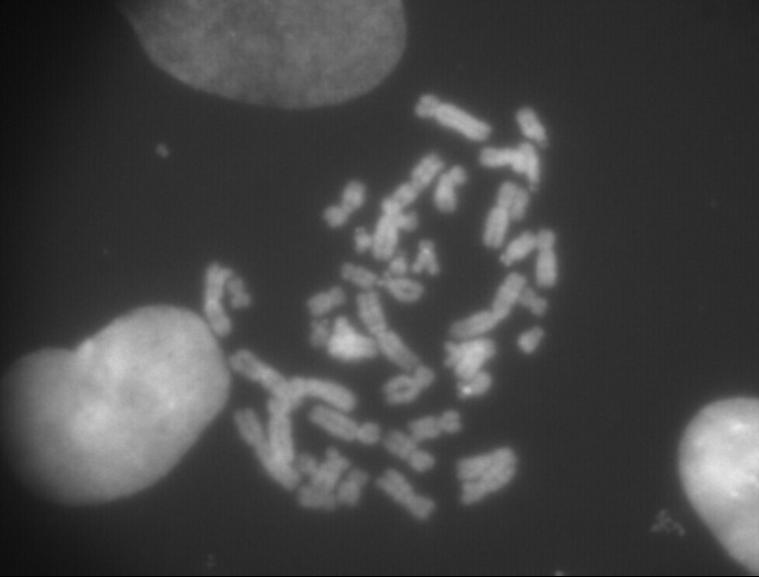

Supplement: Supplementary file 1 [file ijms-26-08886-s001.zip › Supplementary Figure 12 raw DAPI of Figure 5B.tif]

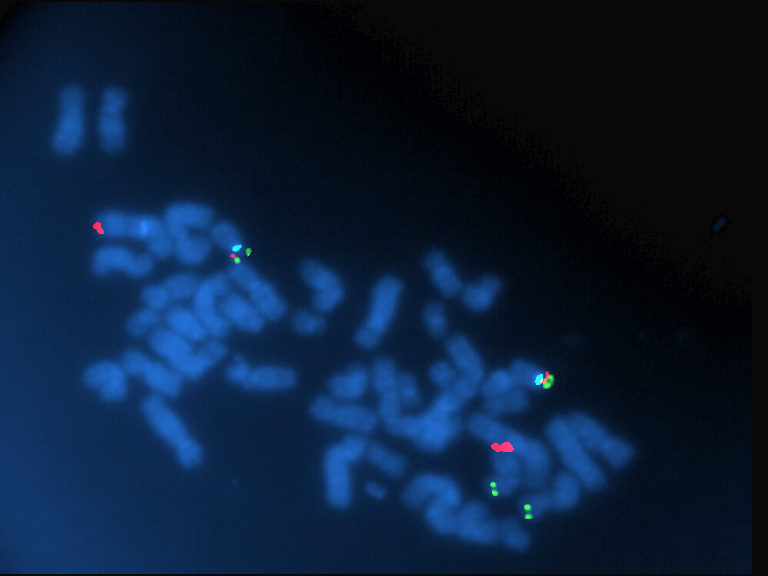

Supplement: Supplementary file 1 [file ijms-26-08886-s001.zip › Supplementary Figure 13.tif]

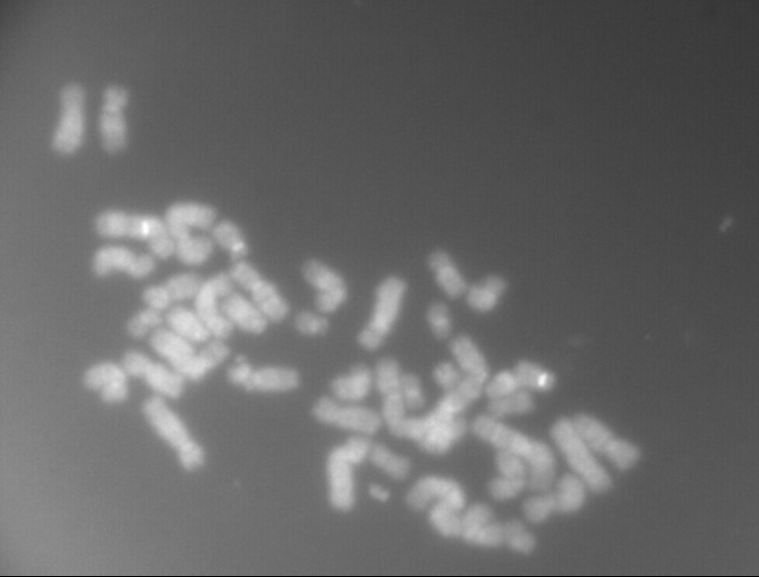

Supplement: Supplementary file 1 [file ijms-26-08886-s001.zip › Supplementary Figure 14 raw DAPI of Suppl Fig 13.tif]

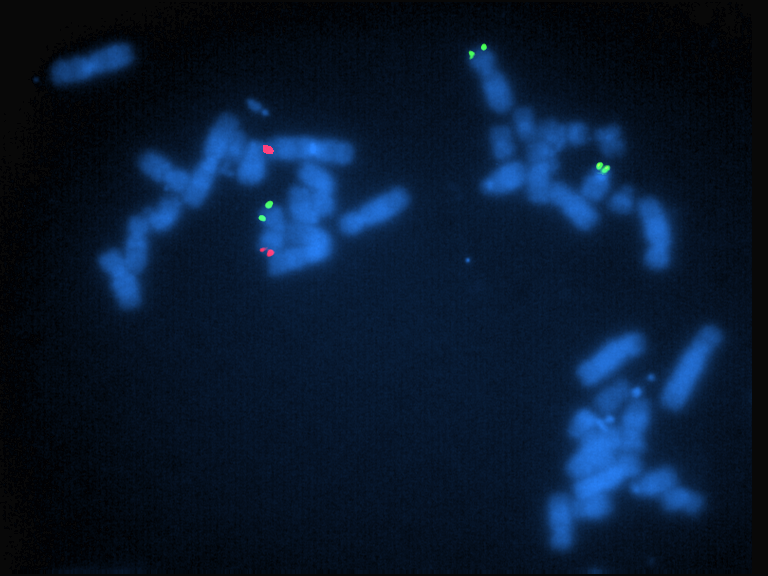

Supplement: Supplementary file 1 [file ijms-26-08886-s001.zip › Supplementary Figure 15.tif]

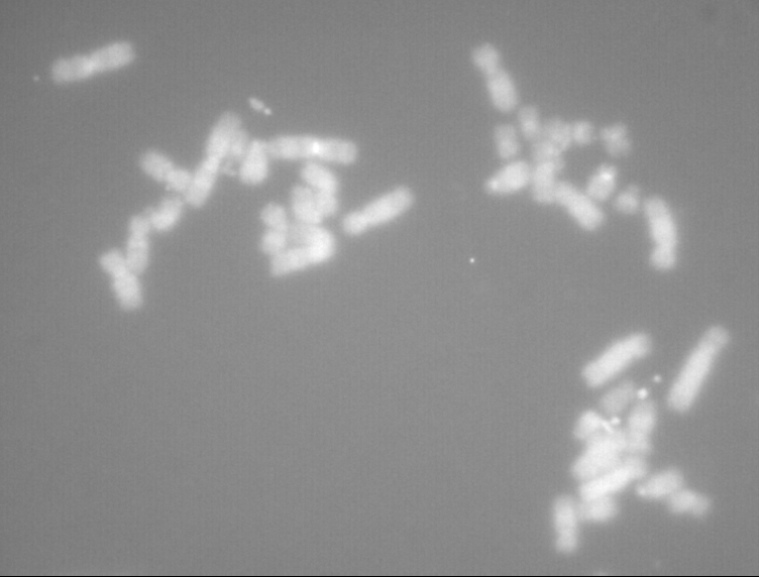

Supplement: Supplementary file 1 [file ijms-26-08886-s001.zip › Supplementary Figure 16 raw DAPI of Suppl Fig 15.tif]

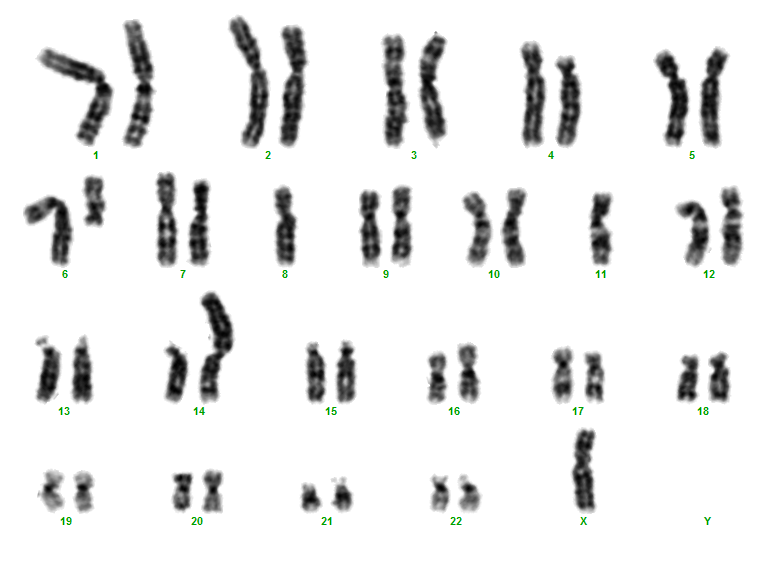

Supplement: Supplementary file 1 [file ijms-26-08886-s001.zip › Supplementary Figure 2.tif]

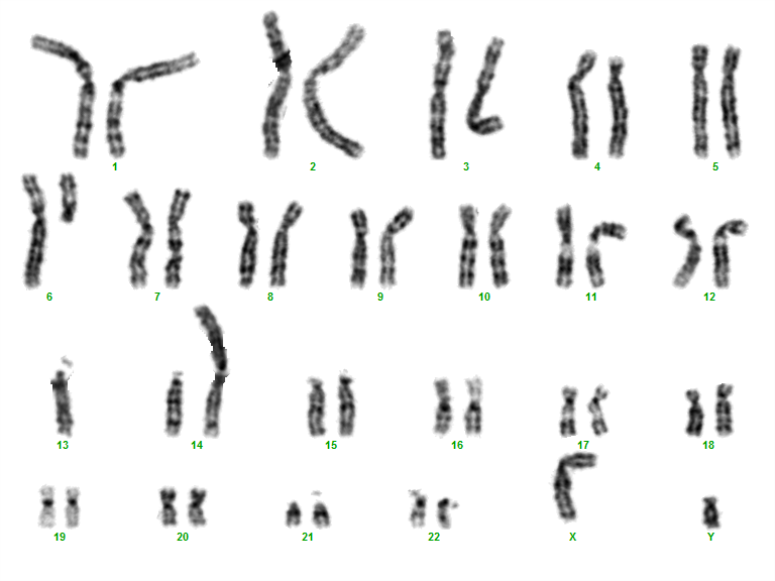

Supplement: Supplementary file 1 [file ijms-26-08886-s001.zip › Supplementary Figure 3.tif]

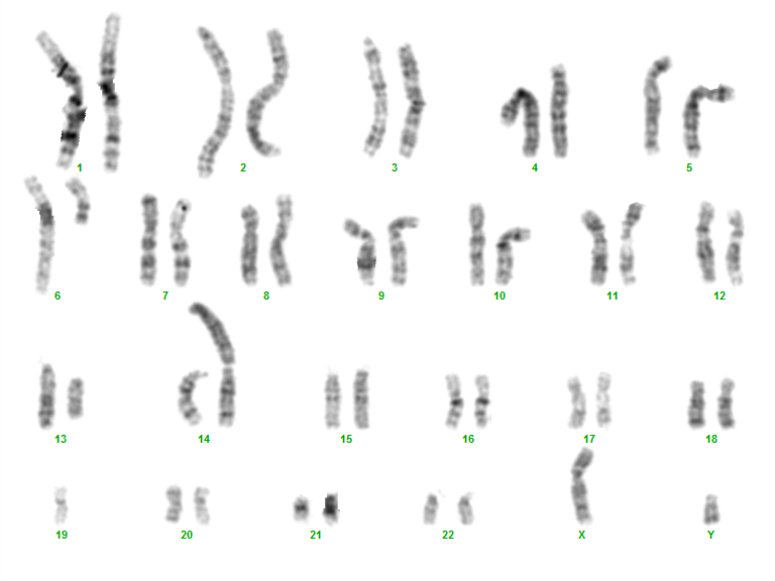

Supplement: Supplementary file 1 [file ijms-26-08886-s001.zip › Supplementary Figure 4.tif]

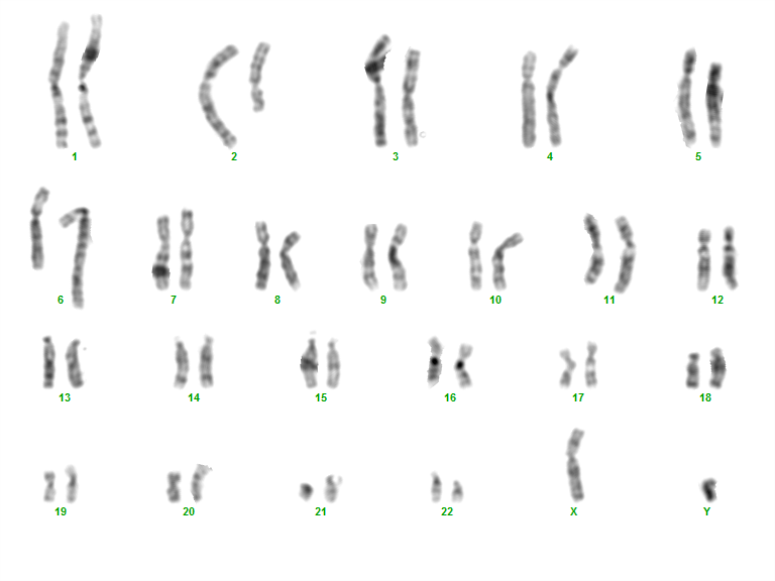

Supplement: Supplementary file 1 [file ijms-26-08886-s001.zip › Supplementary Figure 5.tif]

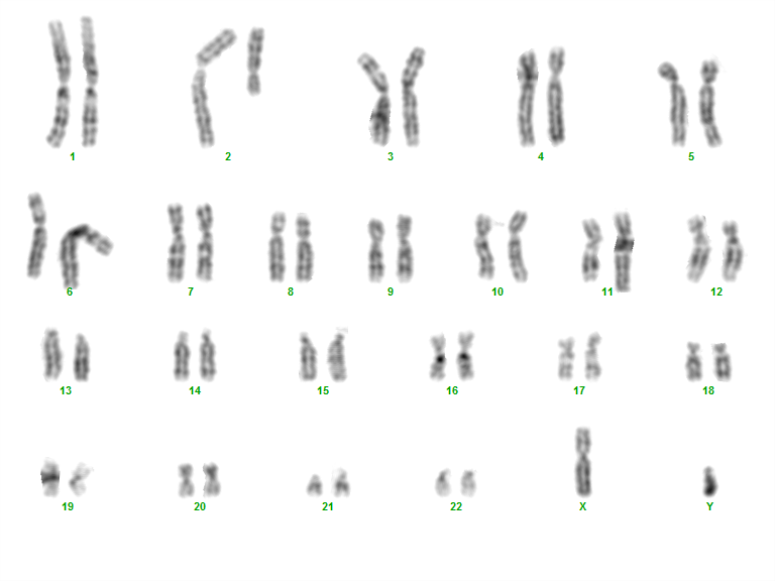

Supplement: Supplementary file 1 [file ijms-26-08886-s001.zip › Supplementary Figure 6.tif]

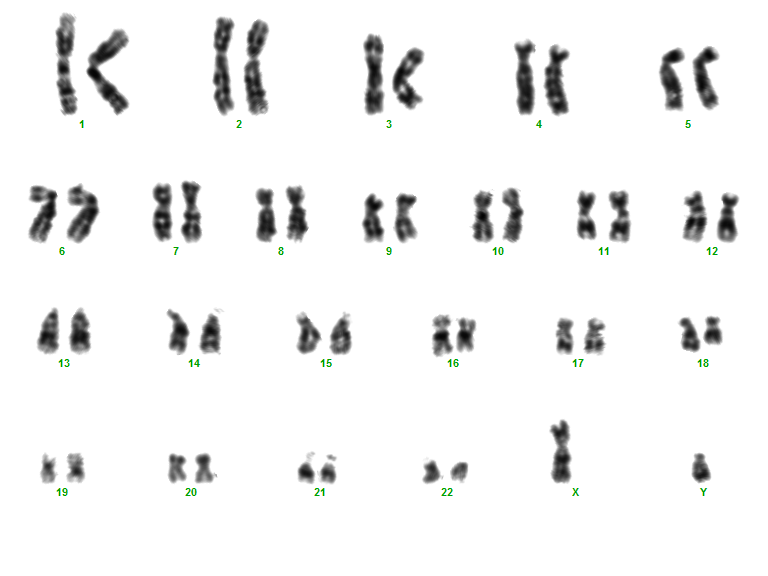

Supplement: Supplementary file 1 [file ijms-26-08886-s001.zip › Supplementary Figure 7.tif]

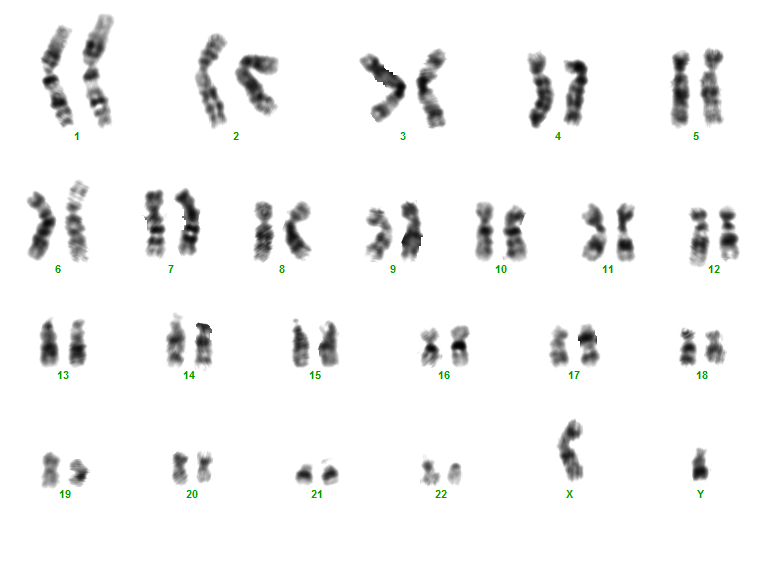

Supplement: Supplementary file 1 [file ijms-26-08886-s001.zip › Supplementary Figure 9.tif]
